# Patient characteristics associated with elective shoulder surgery cancellation: focus on socioeconomic factors

**DOI:** 10.3389/fpubh.2025.1620173

**Published:** 2025-11-12

**Authors:** Suk Woong Kang, Seungwoo Yoon, YunSeo Park, Dayeong Jang, Min Hui Moon, Min Hyeok Choi

**Affiliations:** 1 Department of Orthopedics, Research Institute for Convergence of Biomedical Science and Technology, Pusan National University Yangsan Hospital, Yangsan, Republic of Korea; 2 Department of Orthopedics, School of Medicine, Pusan National University, Yangsan, Republic of Korea; 3 School of Medicine, Pusan National University, Yangsan, Republic of Korea; 4 Department of Preventive and Occupational and Environmental Medicine, School of Medicine, Pusan National University, Yangsan, Republic of Korea; 5 Office of Public Healthcare Service, Pusan National University Yangsan Hospital, Yangsan, Republic of Korea

**Keywords:** shoulder surgery, surgery cancellation, socioeconomic factors, elective surgery, health inequality

## Abstract

**Purpose:**

This study aimed to examine the factors associated with the cancellation of elective shoulder surgeries, focusing mainly on socioeconomic aspects, and to explore these associations stratified by rural and urban areas.

**Methods:**

A retrospective cross-sectional analysis was conducted using the electronic medical records of 1,001 adult patients scheduled for elective shoulder surgery under general anesthesia at a tertiary hospital in South Korea between April 2018 and December 2024. Surgery cancellation was defined as any procedure recorded as “canceled” before the scheduled surgery date. Sociodemographic, clinical, and surgery-related factors were analyzed using chi-squared tests and multivariate logistic regression models. Stratified analyses were also conducted based on residential area.

**Results:**

The overall surgical cancellation rate was 11.5%. Older age (≥65 years), severe disease, rural residence, manual labor, and complex surgical procedures were significantly associated with higher odds of cancellation. The multivariate analysis showed that patients aged 65 years or older (adjusted OR = 2.20, *p* < 0.001), those with severe disease (adjusted OR = 4.29, *p* = 0.004), manual laborers (adjusted OR = 2.93, *p* < 0.001), and rural residents (adjusted OR = 1.87, *p* = 0.006) were at a greater risk of cancellation. Stratified analysis revealed that Medical Aid coverage significantly increased the risk of cancellation in rural areas (adjusted OR = 5.76, *p* = 0.025).

**Conclusion:**

Elective shoulder surgery cancellations are influenced not only by clinical factors but also by socioeconomic and geographical disparities. Patient-centered surgical planning that incorporates individual socioeconomic circumstances is essential for reducing cancellation rates and promoting equitable surgical care.

## 1 Introduction

Delays in elective surgeries can lead to increased healthcare costs owing to prolonged rehabilitation periods and delayed return to work (1). Such delays not only place a financial burden on healthcare systems but also adversely affect patient recovery timelines and overall quality of life. Reducing surgical delays is therefore essential to improving both economic efficiency and patient outcomes. Notably, a study published in the *Annals of Thoracic Surgery* found that in-hospital surgical delays resulted in poorer patient outcomes and increased the financial burden, with an estimated additional cost of approximately USD 3.5 million (2). Specifically, delayed operating room availability and resource constraints were found to prolong hospital stays and contribute to higher rates of complications, including infections and readmissions. These findings highlight that even short-term delays can trigger cascading clinical and financial consequences, emphasizing the importance of timely surgical care and efficient perioperative management.

Delays in elective shoulder surgery due to pre-operative cancellations can have substantial clinical implications. In particular, postponing surgical intervention for conditions such as rotator cuff tears may allow progressive tendon degeneration, muscle atrophy, and fatty infiltration to advance, all of which are strongly associated with poorer surgical outcomes and diminished long-term functional recovery (3). Kim et al. (4) further reported that delaying surgical repair in patients with concomitant shoulder stiffness resulted in less favorable functional outcomes and slower post-operative recovery compared to early intervention (4). Moreover, prolonged symptom duration may contribute to chronic pain and reduced quality of life, with some patients developing central sensitization or persistent pain syndromes that complicate post-operative pain management (4, 5). Dunn et al. also highlighted that pain severity does not always correlate with the anatomical extent of rotator cuff tears, suggesting that postponing surgery based solely on symptom intensity may risk disease progression and worse prognosis (5).

Several studies have investigated factors associated with the cancellation of elective shoulder surgeries. Kim et al. reported that approximately 15% of patients scheduled for elective shoulder surgery at a tertiary medical center in South Korea ultimately canceled their procedures. The most frequently cited reasons included worsening of the patient's medical condition, personal circumstances, and fear of surgery. These findings highlight the importance of appropriate pre-operative education and psychological support to reduce cancellation rates (6, 7).

Kaufman et al. demonstrated that high upfront hospital charges posed a substantial barrier to surgical access for low-income patients, often resulting in cancellations or delays in necessary care (8). Similarly, Greenup emphasized the concept of “financial toxicity,” highlighting that treatment-related costs can drive patients to delay or forego recommended interventions, underscoring the critical role of integrating cost discussions into shared decision-making (9). Together, these findings illustrate how financial barriers can profoundly impact surgical decision-making and patient outcomes, reinforcing the need for systemic solutions to reduce economic disparities in healthcare access.

Therefore, this study aimed to analyze the predictive factors associated with the cancellation of elective shoulder surgery, with a particular focus on socioeconomic factors. Furthermore, we sought to stratify the analysis by rural and urban settings to examine the factors influencing surgical cancellations.

## 2 Methods

### 2.1 Data and study population

This retrospective cross-sectional study was conducted using electronic medical records (EMR) from Pusan National University Yangsan Hospital, South Korea. The study population consisted of adult patients scheduled for elective orthopedic surgery requiring general anesthesia between April 2018 and December 2024. Patients were excluded if they underwent emergency surgery, were transferred to another hospital, or had incomplete records of essential surgical information. After applying these criteria, 1,001 patients were included in the final analysis.

### 2.2 Variables

The primary outcome variable in this study was surgery cancellation, which was defined as any scheduled surgery recorded as “canceled” in the hospital system prior to the planned surgery date. Surgeries that proceeded as scheduled were classified as “performed.”

The independent variables were categorized into sociodemographic, clinical, and surgery-related factors. Sociodemographic factors included sex, age, insurance type [classified as National Health Insurance (NHI) or Medical Aid], occupation (classified as non-manual or manual labor), and residential area (urban or rural). Clinical factors included disease severity, comorbidities, and the type of surgical procedure. Disease severity was assessed clinically and categorized as moderate or severe. Comorbidities were defined as the presence of diabetes mellitus or cardiovascular disease, both of which influence surgical outcomes.

Surgical procedures were grouped into two categories based on clinical complexity and disease characteristics. Group 1 included labral repair, capsular surgery, and simple rotator cuff repair, whereas Group 2 included complex rotator cuff repair and total joint replacement. This classification was intended to reflect the variations in surgical difficulty and clinical burden.

### 2.3 Statistical analysis

Descriptive statistics were used to summarize participants' characteristics. Continuous variables are reported as means and standard deviations, while categorical variables are presented as frequencies and percentages. Associations between surgery cancellation and independent variables were assessed using the chi-squared or Fisher's exact test, as appropriate.

Logistic regression analyses were conducted to identify the factors associated with surgery cancellation. Unadjusted odds ratios (ORs) and 95% confidence intervals (CIs) were determined. Subsequently, multivariate logistic regression models were developed to estimate the adjusted ORs and 95% CIs, controlling for all independent variables. Additionally, stratified logistic regression analyses were performed by residential area (urban vs. rural) to explore the potential differences in influencing factors by region.

All statistical analyses were performed using SAS (Version 9.4; SAS Institute Inc., Cary, NC, USA), with statistical significance set at *p* < 0.05.

### 2.4 Ethical considerations

This study was approved by the Institutional Review Board (IRB) of Pusan National University Yangsan Hospital (IRB No. 05 – 2021-185). The requirement for informed consent was waived due to the retrospective nature of the study and the use of de-identified data. All the research procedures were conducted in accordance with the principles of the Declaration of Helsinki.

## 3 Results

### 3.1 General characteristics of the study population

Table 1 shows the general characteristics of the study population. A total of 1,001 patients were included in this analysis. Among them, 51.7% were male (*n* = 517) and 48.3% were female (*n* = 484). More than half of the patients were younger than 65 years (54.2%, *n* = 542), whereas 45.9% (*n* = 459) were aged 65 years or older.

**Table 1:** General characteristics of the study population.

**Variables**	***N***	**%**
Total	1,001	100.00
**Gender**		
Men	517	51.65
Women	484	48.35
**Age group**		
Under 65	542	54.15
65 year or over	459	45.85
**Types of surgery** ^a^		
Group 1	864	86.31
Group 2	137	13.69
**Area**		
Urban	790	78.92
Rural	211	21.08
**Insurance**		
NHI	942	94.11
Medical aid	59	5.89
**Occupation**		
Non-manual	773	77.22
Manual	228	22.78
**Severity**		
Moderate	979	97.80
Severe	22	2.20
**Diabetes mellitus**		
No	789	78.82
Yes	212	21.18
**Cardiovascular disease**		
No	882	88.11
Yes	119	11.89
**Surgery cancellation**		
Canceled	115	11.49
Performed	886	88.51

^a^Types of surgery: group 1-labral repair, capsular surgery, and simple rotator cuff repair/Group 2-complex rotator cuff repair and total joint replacement.

NHI, National Health Insurance.

In terms of surgical classification, 86.3% of patients (*n* = 864) underwent procedures categorized into the surgical Group 1, including labral repair, capsular surgery, and simple rotator cuff repair. The remaining 13.7% (*n* = 137) underwent Group 2 procedures, comprising complex rotator cuff repair and total joint replacement.

Most patients resided in urban areas (78.9%, *n* = 790), while 21.1% (*n* = 211) lived in rural areas. Most patients were covered by the National Health Insurance, traffic accident insurance, or industrial accident insurance (94.1%, *n* = 942), whereas 5.9% (*n* = 59) were covered by Medical Aid. Regarding occupational status, 77.2% (*n* = 773) were non-manual workers and 22.8% (*n* = 228) were manual laborers. With respect to disease severity, 97.8% (*n* = 979) were classified as having mild to moderate disease and 2.2% (*n* = 22) were considered severe. Comorbid diabetes mellitus was present in 21.2% of the patients (*n* = 212), while 78.8% (*n* = 789) had no diabetes. Additionally, 11.9% (*n* = 119) of the patients had cardiovascular disease, whereas 88.1% (*n* = 882) did not. The overall surgery cancellation rate was 11.5% (*n* = 115), and 88.5% (*n* = 886) of the patients underwent surgery as scheduled.

### 3.2 Surgery cancellation according to patient and clinical factors

The overall surgical cancellation rate was 11.49% (*n* = 115). No significant difference in cancellation rate was observed between men (11.80%) and women (11.16%; *p* = 0.750; Table 2).

**Table 2:** Surgery cancellation rates according to patient and clinical and socioeconomic factors.

**Variables**	***N***	**%**	***p*-Value^b^**
Total	115	11.49	
**Gender**			
Men	61	11.80	0.750
Women	54	11.16	
**Age group**			
Under 65	44	8.12	< 0.001
65 year or over	71	15.47	
**Types of surgery** ^a^			
Group 1	87	10.07	< 0.001
Group 2	28	20.44	
**Area**			
Urban	79	10.00	0.004
Rural	36	17.06	
**Insurance**			
NHI^*c*^	103	10.93	0.028
Medical aid	12	20.34	
**Occupation**			
Non-manual	71	9.18	< 0.001
Manual	44	19.30	
**Severity**			
Moderate	108	11.03	0.009
Severe	7	31.82	
**Diabetes mellitus**			
No	91	11.53	0.931
Yes	24	11.32	
**Cardiovascular disease**			
No	102	11.56	0.837
Yes	13	10.92	

^a^Types of surgery: group 1-labral repair, capsular surgery, and simple rotator cuff repair/Group2 -complex rotator cuff repair and total joint replacement.

^b^Results for chi-squared test or Fisher's exact test.

^c^NHI, National Health Insurance.

Among the examined factors, age, type of surgery, residential area, and insurance type showed statistically significant associations with surgical cancellation. The cancellation rate was significantly higher in patients aged 65 years or older (15.47%) compared to those younger than 65 years (8.12%; *p* < 0.001).

Surgical complexity was also associated with cancellation rate. Patients who underwent procedures classified as Group 2 (complex rotator cuff repair and total joint replacement) had a cancellation rate of 20.44%, which was significantly higher than the rate of 10.07% observed in Group 1 (labral repair, capsular surgery, and simple rotator cuff repair; *p* < 0.001). Regarding residential area, patients living in rural regions experienced a significantly higher cancellation rate (17.06%) than those living in urban areas (10.00%; *p* = 0.004). Similarly, insurance type was also found to be a significant factor. Patients covered by Medical Aid had a notably higher cancellation rate (20.34%) than those covered by National Health Insurance (NHI; 10.93%; *p* = 0.028). In contrast, no significant differences in surgical cancellation rates were observed according to sex, occupation type, disease severity, diabetes mellitus, or cardiovascular disease.

### 3.3 Multivariable analysis of factors associated with surgery cancellation

In the multivariate logistic regression analysis, several factors were significantly associated with surgery cancellation after adjusting for potential confounders (Table 3). Age group remained a significant predictor, with patients aged 65 years or older showing more than twice the odds of surgery cancellation compared to those under 65 years [adjusted OR = 2.20, 95% CI (1.43–3.38), *p* < 0.001]. Surgery type also had a significant association. Patients undergoing complex rotator cuff repair or total joint replacement (Group 2) had a higher risk of cancellation compared to those in Group 1 [adjusted OR = 1.93, 95% CI (1.17–3.20), *p* = 0.010]. Residential area was another significant factor; patients living in rural areas had increased odds of surgery cancellation compared to urban residents [adjusted OR = 1.87, 95% CI (1.20–2.93), *p* = 0.006]. Occupation demonstrated a strong association, with manual laborers exhibiting nearly three times the odds of cancellation compared to non-manual workers [adjusted OR = 2.93, 95% CI (1.87–4.60), *p* < 0.001]. Finally, disease severity was significantly associated with surgical cancellation. Patients classified as severe had markedly higher odds of cancellation than those with moderate disease severity [adjusted OR = 4.29, 95% CI (1.57–11.67), *p* = 0.004]. By contrast, sex, diabetes mellitus, and cardiovascular disease were not significantly associated with surgical cancellation in the adjusted model (all *p* > 0.05). Regarding insurance type, in the unadjusted model, patients with medical aid showed a significantly higher likelihood of surgery cancellation. Although the odds remained higher in the adjusted model, the association was not statistically significant. No baseline characteristics had a VIF greater than 2, indicating low multicollinearity among the included variables. The model demonstrated a pseudo *R*^2^ of approximately 0.0565 (Cox and Snell) and 0.1109 (Max-rescaled Nagelkerke), with an AIC of 675.68.

**Table 3:** Unadjusted and adjusted odds ratios for factors associated with surgery cancellation using logistic regression analysis.

**Variables**	**Unadjusted model**		**Adjusted model** ^**a**^	
	**OR**^b^ **(95% CI**^c^**)**	***p*** **-Value**	**OR (95% CI)**	***p*** **-Value**
**Gender**				
Men	1.07 (0.72–1.57^c^)	0.750	0.97 (0.64–1.49)	0.903
Women	reference		reference	
**Age group**				
< 65	reference		reference	
≥65	2.07 (1.39–3.09)	< 0.001	2.20 (1.43–3.38)	< 0.001
**Types of surgery** ^d^				
Group 1	reference		reference	
Group 2	2.29 (1.43–3.67)	0.001	1.93 (1.17–3.20)	0.010
**Area**				
Urban	reference		reference	
Rural	1.85 (1.21–2.84)	0.005	1.87 (1.20–2.93)	0.006
**Insurance**				
NHI^e^	reference		reference	
Medical aid	2.08 (1.07–4.05)	0.031	1.56 (0.73–3.35)	0.253
**Occupation**				
Non-manual	reference		reference	
Manual	2.36 (1.57–3.56)	< 0.001	2.93 (1.87–4.60)	< 0.001
**Severity**				
Moderate	reference		reference	
Severe	3.76 (1.50–9.44)	0.005	4.29 (1.57–11.67)	0.004
**Diabetes mellitus**				
No	reference		reference	
Yes	0.98 (0.61–1.58)	0.932	0.81 (0.48–1.35)	0.409
**Cardiovascular disease**				
No	reference		reference	
Yes	0.94 (0.51–1.73)	0.837	0.77 (0.40–1.48)	0.431
Cox and Snell *R*^2^			0.0565	
Pseudo *R*^2^			0.1109	
AIC^f^			675.68	

^a^Adjusted for gender, age group, types of surgery, area, insurance, occupation, severity, diabetes mellitus, and cardiovascular disease. ^b^OR, odds ratio. ^c^CI, confidence interval. ^d^Types of surgery: group 1-labral repair, capsular surgery, and simple rotator cuff repair/Group 2-complex rotator cuff repair and total joint replacement. ^e^NHI, National Health Insurance. ^f^AIC, Akaike Information Criterion.

### 3.4 Comparison of factors associated with surgery cancellation according to residential area

Table 4 presents the results of the stratified logistic regression analysis examining the factors associated with surgery cancellation according to the residential area.

**Table 4:** Stratified logistic analysis^a^ of factors associated with surgery cancellation by residential area.

**Variables**	**Urban**		**Rural**	
	**OR**^b^ **(95% CI**^c^**)**	***p*** **-Value**	**OR**^b^ **(95% CI**^c^**)**	***p*** **-Value**
**Gender**				
Men	1.25 (0.75–2.08)	0.393	0.65 (1.20–6.82)	0.325
Women	reference		reference	
**Age group**				
< 65	reference		reference	
≧65	2.18 (1.31–3.63)	0.003	2.86 (1.20–6.82)	0.018
**Types of surgery** ^d^				
Group 1	reference		reference	
Group 2	2.28 (1.24–4.19)	0.008	1.33 (0.52–3.43)	0.551
**Insurance**				
NHI^e^	reference		reference	
Medical Aid	1.08 (0.40–2.86)	0.885	5.76 (1.25–26.66)	0.025
**Occupation**				
Non-manual	reference		reference	
Manual	3.19 (1.89–5.40)	< 0.001	2.29 (0.91–5.80)	0.080
**Severity**				
Moderate	reference		reference	
Severe	4.66 (1.53–14.25)	0.007	10.91 (0.58–20.65)	0.111
**Diabetes mellitus**				
No	reference		reference	
Yes	0.56 (0.28–1.12)	0.102	1.56 (0.67–3.63)	0.300
**Cardiovascular disease**				
No	reference		reference	
Yes	0.78 (0.34–1.76)	0.544	0.90 (0.30–2.77)	0.860
Cox and Snell *R*^2^	0.0524		0.0932	
Pseudo *R*^2^	0.1095		0.1556	
AIC^f^	489.15		190.15	

^a^Adjusted for gender, age group, types of surgery, area, insurance, occupation, severity, diabetes mellitus, and cardiovascular disease. ^b^OR, odds ratio. ^c^CI, confidence interval. ^d^Types of surgery: group 1-labral repair, capsular surgery, and simple rotator cuff repair/Group 2-complex rotator cuff repair and total joint replacement. ^e^NHI, National Health Insurance. ^f^AIC, Akaike Information Criterion.

Age was significantly associated with surgery cancellation in both urban and rural populations. Patients aged 65 years or older had higher odds of cancellation compared to those younger than 65 years in both urban [adjusted OR = 2.18, 95% CI (1.31–3.63), *p* = 0.003] and rural [adjusted OR = 2.86, 95% CI (1.20–6.82), *p* = 0.018] areas.

Among urban patients, several additional factors were significantly associated with surgical cancellation. Patients undergoing complex surgical procedures (Group 2) had higher odds of cancellation than those in Group 1 [adjusted OR = 2.28, 95% CI (1.24–4.19), *p* = 0.008]. Additionally, manual workers exhibited significantly greater odds of cancellation compared to non-manual workers [adjusted OR = 3.19, 95% CI (1.89–5.40), *p* < 0.001]. Patients with severe disease also demonstrated a substantially increased risk of surgery cancellation [adjusted OR = 4.66, 95% CI (1.53–14.25), *p* = 0.007].

By contrast, among rural patients, insurance type was the only factor significantly associated with surgery cancellation. Specifically, patients covered by Medical Aid had a markedly higher risk of cancellation compared to those with National Health Insurance [adjusted OR = 5.76, 95% CI (1.25–26.66), *p* = 0.025].

No significant associations were observed for sex, diabetes mellitus, or cardiovascular disease in either the urban or rural population.

## 4 Discussion

This study investigated the factors associated with the cancellation of elective shoulder surgeries with a particular focus on socioeconomic factors. The overall cancellation rate was 11.5%, which is comparable to or slightly lower than that reported in previous studies. The primary reasons included health deterioration, personal circumstances, and fear of surgery. Our findings support these previous results and further suggest that cancellations of elective surgeries are influenced not only by clinical factors, but also by patients' socioeconomic backgrounds (10).

Older age and disease severity were both significantly associated with higher rates of elective shoulder surgery cancellation. Patients aged 65 years or older had more than double the odds of cancellation compared to younger individuals, consistent with the notion that older adults may face increased physical vulnerability, greater concern about post-operative complications, and limited social support. Additionally, patients with severe underlying conditions were significantly more likely to cancel surgery, with an adjusted odds ratio of 4.29. These findings suggest that both age-related factors and clinical complexity play substantial roles in surgical decision-making (11).

Our findings align with previous studies reporting that older patients are more prone to pre-operative anxiety, post-operative complications, and slower functional recovery, factors that can increase their likelihood of canceling or postponing surgery (12, 13). Tan et al. (14) further demonstrated that comorbidities such as congestive heart failure, advanced chronic kidney disease, and hip fractures were strong independent predictors of last-minute cancellations, with over 60% of cancellations deemed potentially preventable (14). In response to these challenges, Umeno et al. (15) (2022) showed that implementing multidisciplinary pre-operative clinics significantly reduced cancellation rates by improving medical optimization, risk assessment, and care coordination (15). Similarly, Mahure et al. (16) found that diabetes was associated with increased perioperative complications in elective total shoulder arthroplasty, underscoring the importance of comprehensive risk communication and individualized perioperative planning (16). Together, these studies highlight the need for proactive, patient-centered perioperative care pathways tailored to older and medically complex patients to enhance surgical adherence and outcomes.

Patients residing in rural areas and those engaged in manual labor also exhibited significantly higher rates of elective shoulder surgery cancellations. Rural residents had nearly twice the odds of cancellation compared with urban residents, and manual laborers had almost three times the odds compared with non-manual workers. These findings suggest that structural and socioeconomic barriers, such as limited access to healthcare facilities, transportation challenges, financial constraints, and concerns over post-operative work limitations, may disproportionately affect these populations.

Rural patients often face geographical and logistical hurdles that complicate pre-operative assessment and post-operative care (17). Prior studies have indicated that rural residency is associated with lower surgical utilization rates and worse post-operative outcomes, largely because of disparities in healthcare infrastructure and follow-up care access (18–20). Furthermore, manual laborers may be hesitant to undergo elective surgery due to concerns over prolonged absence from work, income loss, and job insecurity, particularly when post-operative rehabilitation is lengthy. Hah et al. demonstrated that post-operative complications and delayed return to work impose substantial socioeconomic burdens, with productivity losses averaging over USD 13,000 per patient (21). Similarly, Jack et al. reported that patients awaiting surgery frequently experience significant financial and emotional hardship, factors that can influence their decision to delay or forgo surgical treatment (22).

Insurance type also played a critical role in elective surgery cancellation, particularly among patients covered by Medical Aid. While insurance type was not a significant predictor in the overall adjusted model, the stratified analysis revealed that among rural patients, those with Medical Aid were nearly six times more likely to cancel surgery than those with National Health Insurance (23). This finding suggests that financial vulnerability and systemic inequities may have a more pronounced effect on medically underserved populations, particularly in rural settings.

Medical Aid recipients often face indirect costs related to surgery, such as transportation, caregiving responsibilities, and loss of daily income, which are not fully offset by insurance coverage (24). Moreover, these patients may have limited health literacy or a distrust of medical systems, leading to greater hesitation in proceeding with elective procedures (25, 26). Awan et al. (27) reported that women with public insurance and those living in socioeconomically deprived neighborhoods experienced significantly longer delays in initiating breast cancer treatment, highlighting the compounding effects of insurance status and structural disparities on timely access to surgical care (27).

The importance of patient-centered surgical planning lies in its ability to align medical decision-making with individual values, preferences, and circumstances of patients, especially those facing socioeconomic or geographic barriers. By actively involving patients in the surgical planning process and considering factors such as occupation, financial burden, and support systems, healthcare providers can improve patient satisfaction, reduce cancellation rates, and enhance surgical outcomes (28). Patient-centered approaches have been shown to increase treatment adherence and foster trust between patients and providers, which is particularly critical in elective procedures where hesitation and uncertainty are common (29, 30).

This study had several limitations. First, the study was conducted at a single tertiary medical center in South Korea, which may limit the generalizability of the findings to other regions or healthcare systems. Second, the retrospective cross-sectional design inherently restricts causal inferences, and some unmeasured confounding variables might have influenced the results. Third, the study relied solely on electronic medical records, which might not have fully capture patient-reported reasons for surgery cancellation, such as psychological concerns, family influences, and cultural beliefs. Additionally, factors such as health literacy, anxiety levels, and previous surgical experiences were not assessed, but could have significantly impacted cancellation decisions. Finally, the sample size for certain subgroups, such as patients with severe disease or Medical Aid recipients in rural areas, was relatively small, which might have limited the statistical power of the subgroup analyses.

Future research should consider prospective multicenter studies incorporating qualitative data to better understand patient decision making and develop targeted interventions.

## 5 Conclusion

This study comprehensively analyzed various factors influencing the cancellation of elective shoulder surgeries and identified older age, severe comorbidities, rural residence, manual labor occupations, and Medical Aid coverage as significant risk factors. To address these issues, patient-centered surgical planning that considers each patient's socioeconomic circumstances should be integrated into the surgical decision-making process.

## Data Availability

The data analyzed in this study is subject to the following licenses/restrictions: Access Limitation: The data from Pusan National University Yangsan Hospital's electronic medical records (EMR) is restricted to authorized personnel and is not publicly available due to privacy and confidentiality concerns. Requests to access these datasets should be directed to Suk Woong Kang, redmaniak@naver.com.
